# Effect on nutritional quality changes in fresh and canned *Scomber australasicus* and *Scomberomorus guttatus*

**DOI:** 10.1002/fsn3.3781

**Published:** 2023-10-21

**Authors:** Ali Aberoumand, Farideh Baesi

**Affiliations:** ^1^ Department of Fisheries Behbahan Katam Alanbia University of Technology Behbahan Iran

**Keywords:** canned fishes, nutritional values, proximate composition, *S. australasicus*, *S. guttatus*

## Abstract

The purpose of present research was to investigate the effect of different storage periods on nutrients composition of the two the fish species. In the present study, proximate composition, energy values and oil indexes of two the fish species *Scomber australasicus* and *Scomberomorus guttatus* were determined. The lipid content of the fresh *S. guttatus* was found significantly higher than the *S. australasicus* (*p* < .05). The *S. australasicus* after 2 months storage found lowest calorie value (275.5 kcal/kg). The calorie values of *S. australasicus* after 4 months storage was 292.5 kcal/kg and for *S. guttatus* after 6 months storage (375.70 kcal/kg) found highest. The number of acidic and peroxide for both types of fish after 2 months storage found 2.66 mg KOH/g and 4.22 meqO_2_/kg for *S. australasicus*, and found 2.47 mg KOH/g and 3.76 meqO_2_/kg for *S. guttatus* which were lowest compared to the other two treatments. The storage periods after the canning process led to a decrease in protein and moisture contents and increase in the lipid content of the canned tuna. The calorie level was related to the parameters such as fat level of the processed *S. guttatus* after 6 months storage which lead to high calorie level (375.70 kcal/kg^−1^), while processed *S. australasicus* after 2 months storage with lower fat content (15.1%) had lower energy value (275.5 kcal/kg^−1^). The highest acidic, and peroxide value for treatment 6 months storage for canned fish *S. guttatus* was 4.34 ± 0.36 mg KOH/g, and 5.74 ± 0.25 (meqO_2_/kg), while for *S. australasicus* was 4.21 ± 0.34 mg KOH/g, and 6.67 ± 0.23 (meqO_2_/kg), It can be concluded for fish 6 months storaged *S. guttatus*, shelf life increased the and can be stored for several years.

## Graphical abstract

The results in the Figure in below showed that *S. guttatus* fish with higher content of energy (Kcal/100 g) and lower content of fish oil indexes such as iodine number and peroxide number, the shelf life of canned fish for 6 months was higher.

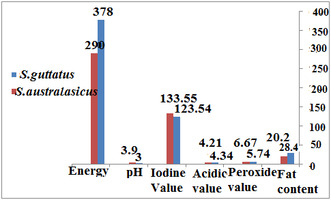

## 1 INTRODUCTION

*Scomber australasicus* and *Scomberomorus guttatus* are very important fish species in Iran, and it have high prices in the market. Most of the tuna caught is for canning, so it is important to study the canning process in order to optimize the nutritional quality of the final product.

Fish can act not only as a source of protein to human being, but also provide foreign exchange earning to many people when the harvesting, handling and processing methods done in the right way and time. In addition, preservation and processing can assure availability of fish in all year round. The bio‐chemical composition of fish is the vital aspect in fish processing, because which influences both the quality and technological characteristics of it (Abraha et al., 2018).

In conclusion, traditional thermal processing methods provide acceptable information and well‐tasting products. Despite this, they have many limitations such as long processing time, limited heat penetration and heterogeneous distribution of heat, which can lead to underheating or overheating problems (Ali et al., 2022).

One of the products that have great economic importance in many countries is canned fish, which fish canning is one of the best and most traditional methods of preserving seafood. In it, heat treatment can significantly change the nature of the raw material, which plays an important role in human nutrition. Both enzymes and bacteria must be permanently inactivated as a result of heat treatment. If recontamination does not occur and negative chemical reactions do not occur with the container, canned seafood should be stored for a very long shelf life (Reblová et al., 2022).

The growth of thermophile spores should be prevented at storage temperatures below 41°C (105°F). Changes in food quality during the storage period are caused by changes in physicochemical and microbiological properties that reduce their nutritional value, palatability and safety of canned fish (El Lahamy & Mohamed, 2020).

Lipid oxidation compounds have shown great importance for the food industry. There are major concerns in food technology due to the oxidation of lipids due to the formation of oxidation products. As a result, undesirable flavors are produced and can reduce the nutritional quality and safety of seafood (Aubourg, 2023).

Fish and its products spoil easily if not preserved. The quality of fish and fish products is due to digestive enzymes, oxidation of lipids and microbes that are actively involved in fish spoilage (Ekonomou et al., 2022). Complex changes in proteins and lipids lead to new products that cause physiological and chemical changes. Therefore, it is necessary to understand and minimize the factors influencing fish spoilage by using active preservation techniques to preserve the freshness of fish and fish‐containing products (Hematyar et al., 2019). Excellent food preservation techniques effectively prevent microbial spoilage and extend product shelf life. Many studies have focused on canning and high temperature storage methods for fish preservation. Fish is part of a healthy diet and provides essential components such as proteins, vitamins, polyunsaturated fatty acids and minerals that are essential for healthy growth. Fish is a highly perishable food and its quality during storage is affected by several factors such as enzymatic autolysis, microbial growth and oxidation (Rathod et al., 2021). The aim of this work was effects of canning process on proximate, oil indexes and energy values of the canned samples after 2, 3 and 4 months storage and nutritive evaluation of fresh and canned *S. guttatus* and *S. australasicus*.

## 2 MATERIALS AND METHODS

### 2.1 Samples preparation

The two fish species in present study purchased from the Iran southern waters. Three piece fishes from each two fish species had 30 ± 2 cm in mean length and 1400 ± 20 g in mean weight. Local name common name, scientific name two fish species are Mackerel tuna (common mackerel) *S. australasicus* (Cuvier, 1832), and Gobad fish (Seer fish), *Scomberomorus guttatus* (Bloch & Schneider, 1801) healthy seafood that is eaten by Iran people as fresh and canned.

### 2.2 Handling and preparation of fresh fishes

The 4 kg each *Scomber australasicus* and *Scomberomorus guttatus* was purchased from the Iran local market. All chemical materials used for analysis were of analytical grade. The distilled water was used in this study. After careful visual examination for the freshness, the fresh fish tuna was doused with running water and immediately cleaned of the wastes. The fish fillets were then cut into sizes of 1 kg and directly placed in a clean plastic bag without washing with water. The 30 same brand canned boiled fish species with 2, 4 and 6 months storage after canning obtained from Behbahan market, Iran. The number of replications was triplicate.

Canned fish oil of 2, 4 and 6 months storage from both types of fish was removed from the cans and each was divided into three equal parts for the analysis of quality parameters of acid, peroxide and iodine indices. Fresh fish oil from each two types of fish were separated after measuring with a soxhlet apparatus to analyze the quality indicators of the oil, similar to canned fish oil. The canned fish fillet of 2, 4 and 6 months of storage of both types of fish was removed from the cans of canned fish, as well as fresh meat, separately were divided for the analysis of proximate compounds and pH. Each analysis was performed in three replicates.

### 2.3 Proximate analysis

The moisture content was determined by drying in an electric oven at 105°C. The compounds analyses were carried out in triplicate. The protein content was analyzed by Kjeldahl method. Ash amount was determined by burning 2 g of sample in a muffle furnace for 2 h at 550°C. Lipid content was measured by methods Bligh and Dyer (1959) and Salam and Davies (1994). The extraction of oil was carried out using Soxhlet apparatus. Twenty grams of fillet powder was weighed and was placed in a thimble while 400 mL of ethanol were measured. The total time for the extraction process was 6 h for each sample. The extracted oil was then collected in a flask.

The yield of the fish oil was obtained from the Equation (1):

(1) Yield=Woil–Wsample/Wsample×100.

Carbohydrates were calculated using the following formula:

$$\text{Carbohydrates}\;\left(\%\right)=100–\left(\text{moisture}+\text{lipids}+\text{proteins}+\mathrm{ash}\right)$$

and the calorie evaluation was done by multiplying the protein, and fat by factors 4.4 and 9 respectively.

### 2.4 Determination of pH

A 10 g sample of the fish sample was homogenized in 100 mL of distilled water and the mixture was filtered. The pH of the filtrate was measured using a pH meter a 713Metrohm digital (Fan et al., 2008).

### 2.5 Determination of acidic value

Three grams of each sample of cooled oil is weighed in 250 mL of conical flasks and 30 mL of neutral ethanol is added to the samples and then shaken well to dissolve the sample. The sample solution was boiled and cooled for about 5 min and then 1 mL of phenolphthalein index was added to the sample solution. The sample was treated with sodium nitrogen 1 hydroxide solution until a light pink color appeared. The acid value was estimated using the following equation:

Acid value=2.82×34𝑉×100/34𝑊×1000×4,

where *W* is weight of oil that equals 3 g, *V* is titer value of 1 N NaOH, and 2.82 is equivalent weight of oleic acid (Food Analysis, 1984).

### 2.6 Determination of peroxide value

Five grams of extracted oil sample from canned or fresh fish fillets by Soxhlet method (AOAC, 2005) was weighed in 250 mL of conical flask; then, 30 mL of acetic acid and chloroform mixture (3:2) was added to oil sample and shaken to dissolve. Then, 1 mL of potassium iodide solution was added to the solution. The solution was kept for 1 min in dark room by shaking occasionally and then 30 mL of distilled water was added. Slowly, titrate iodine in 0.01 N sodium thiosulfate solution until yellow color was gone and after that 1 mL of starch solution indicator was added and we continued titration by shaking to release all I_2_ from CH3Cl layer until blue color disappeared. The peroxide value was estimated using the following equation:

Peroxide value=34𝑉×34𝑁×100/34𝑊,

where *V* is volume of sodium thiosulfate, *N* is normality used for titration, and *W* is weight of the sample (AOAC, 2005).

### 2.7 Determination iodine value

Five grams of oil sample was weighed in 250 mL conical flasks and then 25 mL of carbon tetra chloride was added to oil sample and content was mixed well. 25 mL of Hanus reagent was added to the solution, and then the solution was shaked for proper mixing, and kept in dark for half an hour. After standing, 15 mL of potassium iodide solution was added and then 100 mL of distilled water was added into the mixture and 1 mL starch indicator solution was added to the sample solution. Then, liberated iodine was titrated with 0.01 N of sodium thiosulfate solution; then, at the end, blue color was formed and then disappeared after thorough shaking. The blank determination was carried in the same manner as test sample but without oil. The iodine value was estimated using the following formula:

Iodine value=(34𝑏−34𝑏𝑎)×34𝑁×1.269×100/34𝑊,

where *B* is blank titer value, *A* is sample titer value, *N* is normality of thiosulfate, and *W* is weight of sample (AOAC, 2000).

### 2.8 Statistical analysis

All analysis were done in triplicate, and data averages were calculated using the software SPSS (version 18). The statistical difference between the means, were compared with Duncan test in the significance level (*p* < .05).

## 3 RESULTS AND DISCUSSION

### 3.1 Proximate content

**FIGURE 1:**
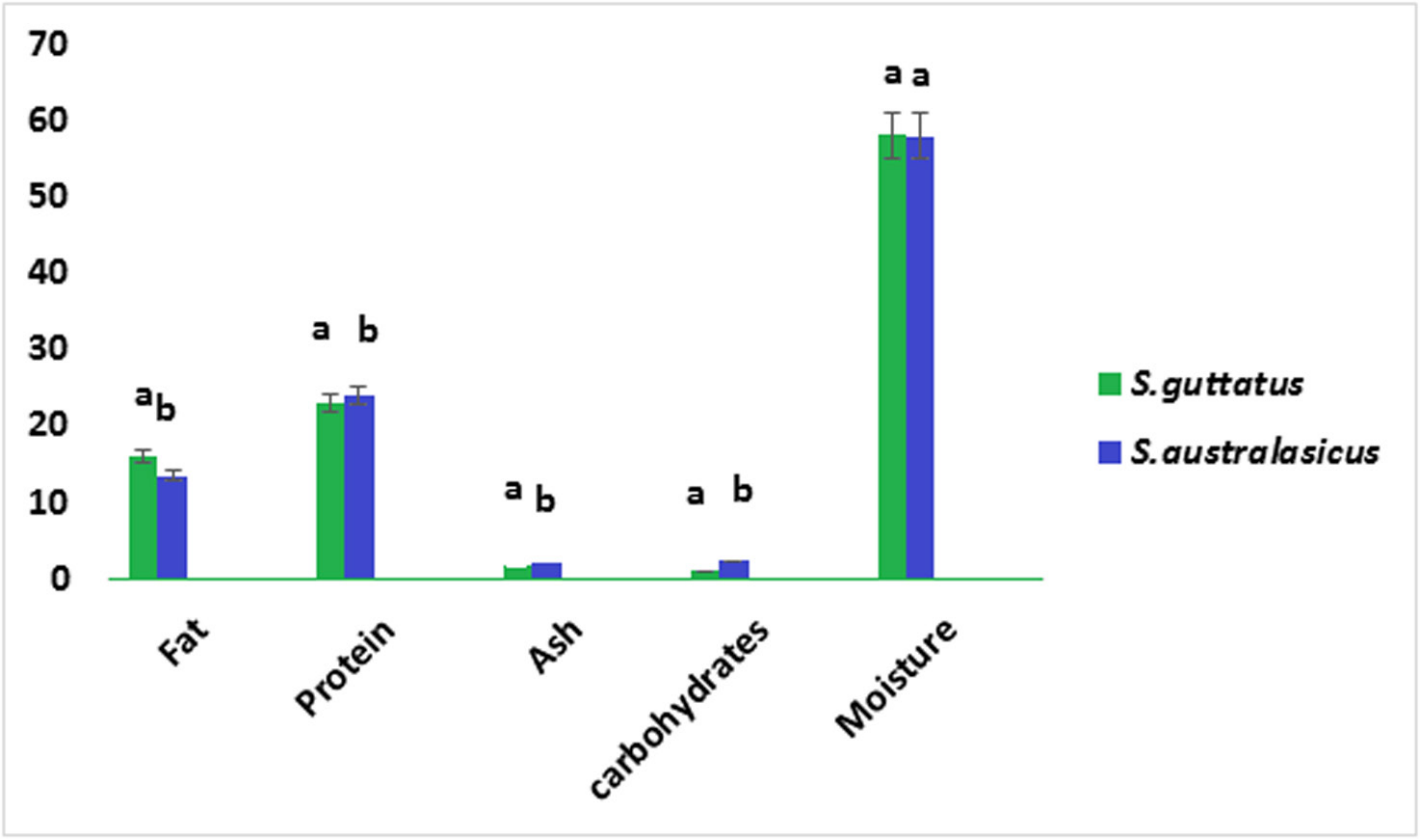
Proximate analysis of fresh *Scomberomorus guttatus* and *Scomber australasicus* fishes. Values represent means ± SD. The different letters on the columns indicate significant differences between the mean data.

**FIGURE 2:**
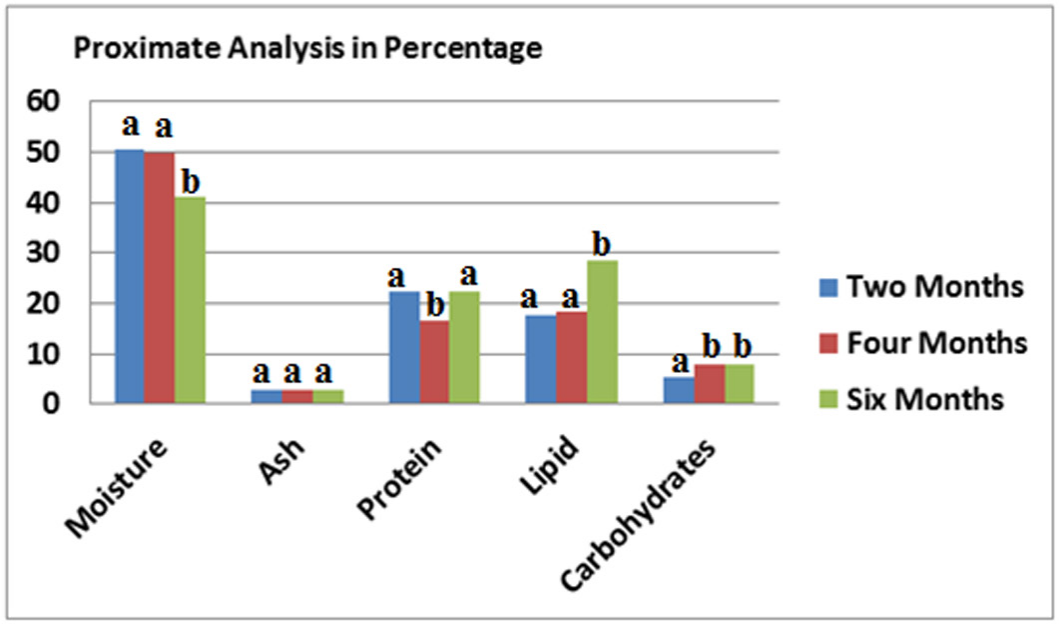
Proximate values of canned *Scomberomorus guttatus* fish after different months of storage Values represent means ± SD. The different letters on the columns indicate significant differences between the mean data.

**FIGURE 3:**
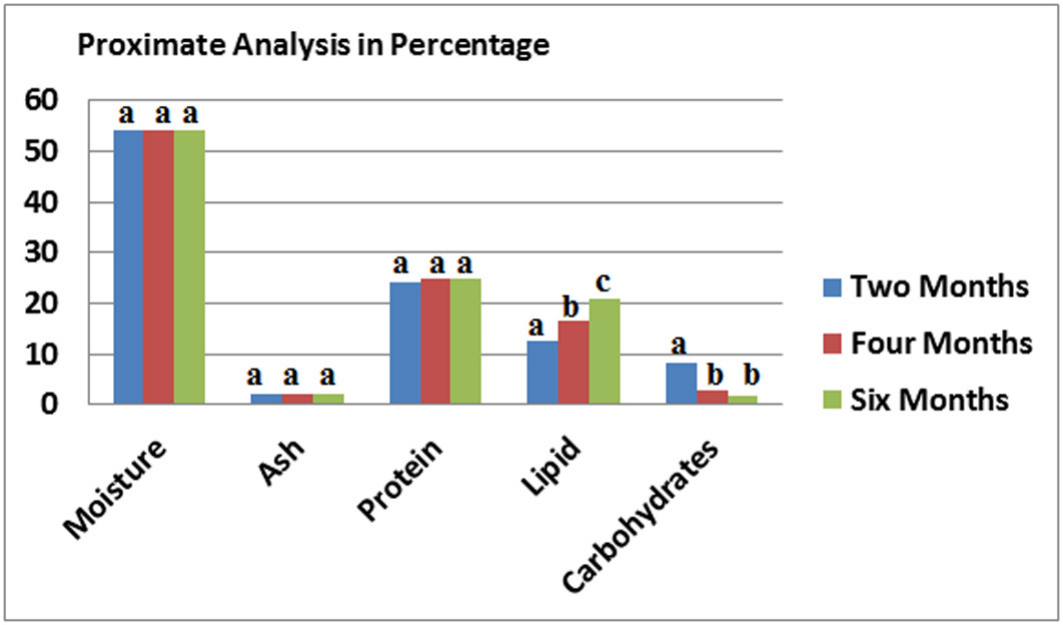
Proximate values of canned *Scomber australasicus* fish after different months of storage. Values represent means ± SD. The different letters on the columns indicate significant differences between the mean data.

The compositions of fresh and processed the samples were shown in Figures 1, 2, 3. With increase in moisture content, the fat amount of fish was decreased. This can be due to the effect of heat in the process, with the effect on interconnection and related changes during process, which reduces the fillet water storage capacity, and thus lead to the fillet loses (Devadasan, 2001). Suresh Kumar (1984) also reported results were agreed with decreasing moisture content ranging from 73.55% at initial stage and 71.20% at the end of 90 days of period. Hence, with decreased moisture contents in 2, 4 and 6 months processed *S. guttatus* sample found 50.6%, 49.9% and 41.3% respectively (Figure 2) also found low fat amount 17.5%, 18.3% and 28.4% respectively. The water content in tuna generally decreases after the heating process is carried out, from 69.40% in fresh tuna to 66.27% in canned tuna. This might be caused by a heating process that occurs during pre‐cooking, exhausting and sterilization. The heat treatment process causes fish to lose water content ranging from 5% to 13%. This is caused by the release of water bound to muscle tissue.

Statistical comparison in terms of differences between peroxide and iodine values in *S. guttatus* and *S. australasicus* for fresh, 2, 4 and 6 months storage showed that there was significant differences (*p* < .05), while *S. guttatus* found better quality. Statistical comparison in terms of differences between acidic value in *S. guttatus* and *S. australasicus* for fresh, 2, 4 and 6 months storage showed that there was not significant differences (*p* < .05).

This increase in fat content can lead to a decrease in moisture content. Agree observations were found by Santha Kumar (2004) for processed in can Tilapia, Peddanna (2005) for squid and shrimp processed in can in masala, Bindu et al. (2007) for black clam and Maheswara (2009) for processed tuna. The protein content found quite high; the highest found in processed *S. australasicus* (24.1%) (Figure 3) in processed sample after 2 months storage. This increase in protein level may be due to a decrease in moisture content during processing and subsequent storage. Similar observations were made by Ramachandran et al. (2010). The canned *S. australasicus* fish contained carbohydrate content of 8.34%, 2.90% and 1.68% after 2, 4 and 6 months storage in room temperature respectively. The highest ash level found in fresh *S. australasicus* fish (2.80%) (Figure 1).

The high protein, fat and ash contents in the canned samples may be because of decrease water by sterilization. Changes in the raw and processed samples pH shown in the Figures 1, 2, 3. It was found that pH of the samples decreased with heat processing (Biji et al., 2015).

The calorie level was related to the fat level of the sample and therefore, processed *S. guttatus* after 6 months storage with high fat amount lead to high calorie level (375.70 kcal/kg^−1^), but processed *S. australasicus* after 1 month storage with lower fat content (15.1%) had lower energy value (275.5 kcal/kg^−1^) (Figure 3).

Due to the advantage of dietary protein, which is important for muscles, both types of fish in the present study had high protein and fat contents (Bonjour, 2005). The fish high fat content leads to high nutritive value as that was affected against heart disease because of fish ω‐3 fatty acids (Alonso et al., 2003). Therefore, it can be concluded that these freshwater fish species are suitable items in a healthy diet. Comparison of nutrient composition between the fresh and processed fish showed that after 3 months of storage in processed samples, fat and total calorie level increased. The level of fat and moisture for any fish varies depending on the season, feeding and catch location, size, etc. Low ash and high protein contents were obtained from proximate analysis showed in Figures 2 and 3 which were in agreed with analysis of Effiong and Mohammed (2008), Mumba and Jose (2005), and Abdullahi (2001).

Protein is a source of amino acids that play an important role as a builder and regulator of the human body. During the sterilization process, about 40% of the total amino acids in fish meat are decomposed (Anwar et al., 2020).

Determining the amount of fat in fish is important. Fish oil contains ω‐3 fatty acids, especially EPA and DHA, which are beneficial for the human body. In food, fat acts as a carrier of vitamins, flavor and as an important source of calories (Anwar et al., 2020).

The peroxide content is an indicator in oil containing unsaturated fatty acids under oxidation. A high amount of peroxide indicates that the oil has been oxidized. Peroxide compounds can accelerate the process of food rancidity and spoilage. The results of the analysis showed that the peroxide value of canned fish ranged from 3.76 to 6.67 meqO_2_/kg with an average value of 5.10 meqO_2_/kg for both fish species (Anwar et al., 2020).

The rancidity in oil is due to the oxidation of fatty acids to form free radicals, so peroxide, an intermediate product, is produced and decomposed to form compounds of aldehydes and ketones. Low PV indicated that the canning process had less effect on the rancidity of the two canned *S. guttatus* fish. This was because at the time of canning fish, the oil used was fresh and still at its best quality and had never gone rancid. Rancidity in food products can reduce the quality of products which is due to the interaction of fat and oxygen, which causes the formation of peroxides that are unstable and easily decompose into aldehydes, ketones (Anwar et al., 2020).

The iodine number can increase which is due to the oxidation of double bonds in fatty acids, which represents the temperature profile of the extraction mixture. Iodine content of 110.51 g/100 g of oil was recorded for oil extracted from mackerel which is not close to the finding of the study as well as to Bako et al. (2017) and present study results, due to fish species, age, nutrition, etc. The peroxide test is used to determine the start of rancidity in oil, but it does not indicate the progression of lipid oxidation. It does not reflect the extent or amount of oil decomposition caused by various oxidative factors and should not exceed 10 meqO_2_/kg which is comparable to the present study results as well as to those of Nazir et al. (2017).

The iodine number of the oil is determined by some reliable laboratory centers. Iodine number in oil samples from the same species of fish can be significantly different. Thus, the oil with the iodine number 140.07 might vary in samples by something like 130–155 (Stansby, 1991). The our study results showed that iodine values found in range minimum 123.54 g I_2_/100 g to maximum 170.45 g I_2_/100 g.

The higher the temperature of sterilization the higher the decrease in water content. Canned tuna after adding the oil medium had a higher fat content than fresh tuna. Increased fat content can be associated with a decrease in moisture content and penetration of oil from the medium into the product. The decrease in protein content is thought to be caused by the occurrence of protein denaturation. The heating process causes heat to penetrate to the fish chunks and reduce the functional properties of the protein. The resistance of protein to heat is strongly related to amino acids constituent. In canned products, the amino acids can be reduced by around 10%–20%. Amino acids in processed foods can be decreased because of its sensitivity to heat. Heat treatment can cause protein to decrease with increasing temperature or the length of the processing time. The loss of protein can be caused by three possible reasons, namely pre‐cooking, diffusion into the liquid and heat destruction during thermal processing. Moreover, increasing the heating time resulted severe protein degradation which lead to the damage offish muscle and fibers. Reduction in protein content can also occur because of the large amount of water contained in a substance (Anwar et al., 2020).

The high protein, fat and ash contents in the canned samples may be because of decrease water by sterilization. Changes in the raw and processed samples pH shown in the Figures 1, 2, 3. It was found that pH of the samples decreased with heat processing (Biji et al., 2015).

The highest acidic value found for oil of six monthly canned fish *S. guttatus* (4.34 ± 0.36 mg KOH/g). The iodine number of oil of two fresh fish species were significantly differences (*p* < .05) (Table 1). The fresh *S. australasicus*ish fish oil iodine number (159.13 ± 4.23 g I_2_/100 g) (Table 2) found higher than *S. guttatus* oil (151.11 ± 4.11 g I_2_/100 g), because levels of unsaturated fatty acids, especially (DHA) in *S. australasicus*ish oil were higher than the *S. guttatus* oil. The work results (Candella et al., 1998; Khoddami et al., 2012) were in agreed with our study results. Endo et al. (2005) reported fish oil iodine number was in the range of 55–188 g/100. The iodine value of fish oil depended on fishing seasons, feeding, and different fish species. Peroxide value is one of the most common indicators of edible oil quality in production, and storage processes, which indicates the degree of oxidation in food (Saad et al., 2006).

**TABLE 1:** Averages of peroxide, acidic, and iodine values of fresh and canned fish *Scomberomorus guttatus.*

Fish species	Peroxide value (meqO_2_/kg)	Acidic value (mg KOH/g)	Iodine value (g I_2_/100 g)
Fresh *S. guttatus*	2.88 ± 0.72^a^	2.55 ± 0.31^a^	151.11 ± 4.11^a^
Two months storage	3.76 ± 0.53^b^	2.47 ± 0.34^a^	162.43 ± 2.46^b^
Four months storage	4.86 ± 0.32^c^	3.62 ± 0.33^b^	141.36 ± 3.46^c^
Six months storage	5.74 ± 0.25^d^	4.34 ± 0.36^c^	123.54 ± 3.56^d^

*Note*: Values represent means ± SD. The different letters on the columns indicate significant differences between the mean data.

**TABLE 2:** Averages of peroxide, acidic, and iodine values of fresh and canned fish *Scomber australasicus.*

Fish species	Peroxide value (meqO_2_/kg)	Acidic value (mg KOH/g)	Iodine value (g I_2_/100 g)
Fresh *S. australasicus*	3.15 ± 0.71^a^	2.67 ± 0.34^a^	159.13 ± 4.23^a^
Two months storage	4.22 ± 0.56^b^	2.63 ± 0.32^a^	170.45 ± 2.45^b^
Four months storage	5.33 ± 0.35^c^	3.57 ± 0.31^b^	151.35 ± 3.43^c^
Six months storage	6.67 ± 0.23^d^	4.21 ± 0.34^c^	133.55 ± 3.41^d^

*Note*: Values represent means ± SD. The different letters on the columns indicate significant differences between the mean data.

PH of fresh fish *S. guttatus* was 5, which decreased to 3 as a result of storage with a significant difference (*p* < .05), which indicated the production of free fatty acids from fish oil, while the energy percentage of canned fish with 6 months of storage was the highest with a significant difference (*p* < .05) (Table 3).

**TABLE 3:** Averages of pH and energy values of fresh and canned fish *Scomberomorus guttatus.*

Fish samples	Fresh *S. guttatus*	Two months storage	Four months storage	Six months storage
PH value	5^a^	3^b^	3^b^	3^b^
Energy value (Kcal)	251.62 ± 6.07^b^	270 ± 6.12^a^	272 ± 6.14^a^	378 ± 6.52^c^

*Note*: Values represent means ± SD. The different letters on the columns indicate significant differences between the mean data.

Table 4 showed that the pH of fresh *S. australasicus* fish also decreased due to storage, but this decrease rate was less than that of *S. guttatus* fish, while the energy percentage of fish with 6 months of storage was the highest with a significant difference (*p* < .05).

**TABLE 4:** Averages of pH and energy values of fresh and canned fish *Scomber australasicus.*

Fish sample	Fresh *S. australasicus*	Two months storage	Four months storage	Six months storage
PH value	5^a^	4^b^	3.9^c^	3.9^c^
Energy value (Kcal)	237.36 ± 6.75^a^	272 ± 6.19^b^	260 ± 5.72^c^	290 ± 6.62^d^

*Note*: Values represent means ± SD. The different letters on the columns indicate significant differences between the mean data.

To control the quality of fish oil, there are several parameters, including the amount of iodine, the amount of peroxide, which determine the quality and thus the economic value of the product (Mendil et al., 2009). The results of a study on the effect of processing methods on fish showed that the canning process is the best process that preserves fish for a long time. According to the conducted study by Aberoumand (2014) lean fish are not recommended to canning process because their flesh disintegrates under the canning with high temperature, hence their delicate flavor and texture would be lost.

Moreover, fish muscle can lose its nutritional composition such as proteins, minerals and vitamins if canning is carried out in oil, since proteins are denatured by the heat process to the point of releasing a considerable amount of water to the headspace of the can. Significance of fish canning is that bones become soft texture and thus edible, providing an important calcium source. On the other hand heat sensitive vitamins, like thiamine, riboflavin, niacin, are the nutrients damaged at the time of sterilization process (Abraha et al., 2018).

## 4 CONCLUSIONS

This study showed basic indexes of fresh and canned two fish species was significantly different. The protein content after 6 months from storage found lower than other samples. It may be concluded that the fish fillet was suitable in the human health diet. It can also be concluded that energy value after 4 months storage in *S. australasicus* was 292.5 kcal kg^−1^ and after 6 months from canning process in *S. guttatus* was 375.70 kcal kg^−1^ which found highest. The peroxide and acidic values of two types of fish after 2 months of storage was the lowest compared to the other two treatments except for fresh fish. Results showed shelf life of the fish two monthly canned found best. Canning process improved shelf life enabling storage of the product for several years; but the processor, nutritionist, and consumer have a direct interest in the composition of fish, as they are all interested in nutritional contribution of the fish to the diet as to translate to good health.

## AUTHOR CONTRIBUTIONS

**Ali Aberoumand:** Formal analysis (equal); funding acquisition (equal); investigation (equal); methodology (equal); visualization (equal); writing – original draft (equal). **Farideh Baesi:** Formal analysis (equal); investigation (equal); methodology (equal).

## FUNDING INFORMATION

This work was supported by Behbahan Khatam Alanbia University of Technology, Behbahan, Iran.

## CONFLICT OF INTEREST STATEMENT

The authors do not have any conflict of interest to declare.

## ETHICS STATEMENT

This study did not involve human or animal testing.

## INFORMED CONSENT

Written informed consent was obtained from all participants.

## Data Availability

Research data are available after request.

## References

[fsn33781-bib-0001] Abdullahi, S. A. (2001). Investigation of nutritional status of *Chrysichthys nigrodigitatus*, *Barus filamentous* and *Auchenoghats occidentals*, family Bangdae. Journal of Arid Zone Fish, 1, 39–50.

[fsn33781-bib-0002] Aberoumand, A. (2014). Studies on the effects of processing on food quantity of two selected consumed marine fishes in Iran. International Food Research Journal, 21(4), 1429–1432.

[fsn33781-bib-0003] Abraha, B. , Admassu, H. , Mahmud, A. , Tsighe, N. , Shui, X. W. , & Fang, Y. (2018). Effect of processing methods on nutritional and physico‐chemical composition of fish: A review. MOJ Food Processing & Technology, 6(4), 376–382.

[fsn33781-bib-0004] Ali, A. , Wei, S. , Ali, A. , Khan, I. , Sun, Q. , Xia, Q. , Wang, Z. , Han, Z. , Liu, Y. , & Liu, S. (2022). Research progress on nutritional value, preservation and processing of fish—A review. Food, 11, 3669. 10.3390/foods11223669 PMC9689683 36429260

[fsn33781-bib-0005] Alonso, A. , Martinez‐Gonzalez, M. A. , & Serrano‐Martinez, M. (2003). Fish ω‐3 fatty acids and risk of coronary heart disease. Medicine Clinical, 121, 28–35. 10.1016/S0025-7753(03)74116-5 12812707

[fsn33781-bib-0006] Anwar, S. H. , Poena, A. A. R. M. , Muzaifa, M. , & Hasan, H. (2020). Canning of traditional acehnese food made by dried little tuna (*Euthynnus affinis*) using two sterilization methods. IOP conferences series: Materials science and engineering 845 012023. IOP Publishing. 10.1088/1757-899X/845/1/012023

[fsn33781-bib-0007] AOAC . (2000). Official method 920.159‐iodine absorption number of oils and fats/food analysis part‐III‐1984 (17th ed.). AOAC.

[fsn33781-bib-0008] AOAC . (2005). Official method 965.33 peroxide value in oils and fats/Pearson's composition and analysis of food (17th ed.). AOAC.

[fsn33781-bib-0009] Aubourg, S. P. (2023). Enhancement of lipid stability and acceptability of canned seafood by addition of natural antioxidant compounds to the packing medium—A review. Antioxidants, 12, 245. 10.3390/antiox12020245 36829804 PMC9952551

[fsn33781-bib-0010] Bako, T. , Umogbai, V. I. , & Awulu, J. O. (2017). Criteria for the extraction of fish oil. Agricultural Engineering International, 19(3), 120–132.

[fsn33781-bib-0011] Biji, K. B. , Shamseer, R. M. , Mohan, C. O. , Ravishankar, C. N. , Mathew, S. , & Gopal, T. K. S. (2015). Effect of thermal processing on the biochemical constituents of green mussel (*Perna viridis*) in Tin‐free‐steel cans. Journal of Food Science and Technology, 52(10), 6804–6809. 10.1007/s13197-015-1757-8 26396433 PMC4573171

[fsn33781-bib-0012] Bindu, J. , Ravishankar, C. N. , & Srinivasa Gopal, T. K. (2007). Shelf life evaluation of a ready‐to‐eat black clam (*Villorita cyprinoids*) product in indigenous report pouches. Journal of Food Engineering, 78, 995–1000. 10.1016/j.jfoodeng.2005.12.040

[fsn33781-bib-0013] Bligh, E. G. , & Dyer, W. J. A. (1959). Rapid method of total lipid extraction and purification. Canadian Journal of Biochemistry and Physiology, 37, 911–917. 10.1139/o59-099 13671378

[fsn33781-bib-0014] Bonjour, J. P. (2005). Dietary protein: An essential nutrient for bone health. Journal of the American College of Nutrition, 24, 526S–536S. 10.1080/07315724.2005.10719501 16373952

[fsn33781-bib-0015] Candella, M. , Astiasaran, I. , & Bello, J. (1998). Deep‐fat frying modifies high fat fish lipid fraction. Journal of Agricultural and Food Chemistry, 46(7), 2793–2796. 10.1021/jf9709616

[fsn33781-bib-0016] Devadasan, K. (2001). Report pouch packaging of fish curry. Fish Curry, 20(10–11), 44–46.

[fsn33781-bib-0017] Effiong, B. N. , & Mohammed, I. (2008). Effects of seasonal variation on the nutrient composition in selected fish species in Lake Kainji‐Nigeria. Nature Science, 6(2), 25–28.

[fsn33781-bib-0018] Ekonomou, S. I. , Parlapani, F. F. , Kyritsi, M. , Hadjichristodoulou, C. , & Boziaris, I. S. (2022). Preservation status and microbial communities of vacuum‐packed hot smoked rainbow trout fillets. Food Microbiology, 103, 103959. 10.1016/j.fm.2021.103959 35082076

[fsn33781-bib-0019] El Lahamy, A. A. , & Mohamed, H. R. (2020). Changes in fish quality during canning process and storage period of canned fish products: Review article. Global Journal of Nutrition & Food Science, 3(1), 1–7. 10.33552/GJNFS.2020.03.000553

[fsn33781-bib-0020] Endo, Y. , Tagiri‐Endo, M. , & Kimura, K. (2005). Rapid determination of iodine value and saponification value of fish oils by near‐infrared spectroscopy. Journal of Food Science, 70(2), C127–C131. 10.1111/j.1365-2621.2005.tb07072.x

[fsn33781-bib-0021] Fan, W. , Chi, Y. , & Zhang, S. (2008). The use of tea polyphenol dip to extend the shelf life of silver carp (*Hypophthalmichthys molitrix*) during storage in ice. Food Chemistry, 108, 148–153. 10.1016/j.foodchem.2007.10.057

[fsn33781-bib-0022] Food Analysis (part III) . (1984). Method of sampling and test for oils and fats/ISO 6601 1996 Determination of acid value and acidity . P. 67/IUPAC2.201 (1979) I.S, 548 (Part‐1).

[fsn33781-bib-0023] Hematyar, N. , Rustad, T. , Sampels, S. , & Kastrup Dalsgaard, T. (2019). Relationship between lipid and protein oxidation in fish. Aquatic Research, 50, 1393–1403. 10.1111/are.14012

[fsn33781-bib-0024] Khoddami, A. , Ariffin, A. A. , Bakar, J. , & Ghazali, H. M. (2012). Quality and fatty acids profile of the oil extracted from fish waste (head, intestine and liver) (*Euthynnus affinis*). African Journal of Biotechnology, 11(7), 1683–1689. 10.5897/AJB10.1699

[fsn33781-bib-0025] Maheswara, K. J. (2009). Studies on thermal processing of tuna in tin can and tin‐free‐steel cans (Master thesis). Karnataka Veterinary, Animal and Fisheries Sciences University, Bidar, India.

[fsn33781-bib-0026] Mendil, D. , Uluozl, O. D. , Tuzen, M. , & Soylak, M. (2009). Investigation of the levels of some element in edible oil samples produced in Turkey by atomic absorption spectrometry. Journal of Hazardous Materials, 165, 724–728. 10.1016/j.jhazmat.2008.10.046 19036503

[fsn33781-bib-0027] Mumba, P. P. , & Jose, M. (2005). Nutrient composition of selected fresh and processed fish species from Lake Malawi, a nutritional possibility for people living with HIV/Aids. International Journal of Consumer Studies, 29(1), 72–77. 10.1111/j.1470-6431.2005.00377

[fsn33781-bib-0028] Nazir, N. , Diana, A. , & Sayuti, K. (2017). Physicochemical and fatty acid profile of fish oil from head of tuna (*Thunnus albacares*) extracted from various extraction method. International Journal Advance Science Engineering Information Technology, 7(2), 709–715. 10.18517/ijaseit.7.2.2339

[fsn33781-bib-0029] Peddanna, V. C. (2005). Modified procedures for canning of shrimp and squid in masala (Master thesis). Karnataka Veterinary, Animal and Fisheries Sciences University, Bidar, India.

[fsn33781-bib-0030] Ramachandran, D. , Mohan, M. , & Sankar, T. V. (2010). Effects of thermal modification on physico‐chemical and functional properties of myofibrillar proteins from Tilapia *Oreochromis mosambicus* (Peters, 1852). Fishery Technology, 47(1), 41–50.

[fsn33781-bib-0031] Rathod, N. B. , Ranveer, R. C. , Benjakul, S. , Kim, S. K. , Pagarkar, A. U. , Patange, S. , & Ozogul, F. (2021). Recent developments of natural antimicrobials and antioxidants on fish and fishery food products. Comprehensive Reviews in Food Science and Food Safety, 20, 4182–4210. 10.1111/1541-4337.12787 34146459

[fsn33781-bib-0032] Reblová, Z. , Aubourg, S. P. , & Pokorný, J. (2022). The effect of different freshness of raw material on lipid quality and sensory acceptance of canned sardines. Food, 11, 1987. 10.3390/foods11131987 PMC9265563 35804805

[fsn33781-bib-0033] Saad, B. , Wai, W. T. , Lim, B. P. , & Saleh, M. I. (2006). Flow injection determination of peroxide value in edible oils using triiodide detector. Analytica Chimica Acta, 565(2), 261–270. 10.1016/j.aca.2006.02.039

[fsn33781-bib-0034] Salam, A. , & Davies, P. M. C. (1994). Body composition of northern pike (*Esox lucius* L.) in relation to body size and condition factor. Journal of Fish Research, 19, 193–204. 10.1016/0165-7836(94)90038-8

[fsn33781-bib-0035] Santha Kumar, G. (2004). Utilization of Tilapia by canning (Master thesis). UAS, Bangalore, India.

[fsn33781-bib-0036] Stansby, M. E. (1991). Fish and fish oil in the diet and its effects on certain medical conditions. National Oceanic and Atmospheric Administration National Marine Fisheries Service Northwest Fisheries Science Center.

[fsn33781-bib-0037] Suresh Kumar, M. (1984). Studies on the canning of pink perch (Master thesis). UAS, Bangalore, India.

